# Extrapontine myelinolysis presenting as acute parkinsonism

**DOI:** 10.1186/1471-2377-6-33

**Published:** 2006-09-10

**Authors:** J Sajith, A Ditchfield, HA Katifi

**Affiliations:** 1 Department of Adult Medicine, Queen Elizabeth Hospital, Woolwich, London. Correspondence to: 82 St Nicholas House, Queen Elizabeth Hospital, Stadium Road, Woolwich, London, SE 18 4QN, UK; 2 Department of Neuroradiology, Southampton General Hospital, Southampton SO9 4XY, UK; 3 Department of Neurology, Southampton General Hospital, Southampton SO9 4XY, UK

## Abstract

**Background:**

Extrapontine myelinolysis presenting with extra pyramidal features suggestive of parkinsonism may be a challenging clinical syndrome. Clinicians should maintain their vigilance while correcting electrolyte imbalances, especially with associated co-morbidity.

**Case presentation:**

A 41-year-old woman presented with acute parkinsonism like features while on a holiday. This followed slow correction of hyponatraemia after repeated vomiting. MRI changes were suggestive of Extrapontine myelinolysis(EPM). This case is at variance with four previous cases reported in the medical literature in that the patient made a full clinical recovery and the MR changes resolved with symptomatic support alone.

**Conclusion:**

Extrapontine myelinolysis could make a complete recovery with symptomatic support alone. During hyponatraemia correction, rapid osmotic shifts of fluid that cause hypernatremia, causes myelinolysis rather than absolute serum sodium level.

Even gradual correction of hyponatraemia can produce myelinolysis, especially with pre-existing malnourishment, alcoholism, drug misuse, Addison's disease and immuno-suppression. Pallidial sparing is typical of EPM in MRI scans.

## Background

Extrapontine myelinolysis presenting with parkinsonism like features may be a challenging clinical syndrome with wide differential including neurodegenerative disease, vascular and metabolic conditions and exposure to toxin (see table 1). We report a case of EPM presenting with extra pyramidal features and florid disability following correction of hyponatraemia and where the patient made a rapid and complete recovery.

## Case presentation

A 41-year-old caucasian woman presented with ataxia, tremor, dysarthria, and cogwheel rigidity. Two weeks prior to admission she had been on a holiday in southwest Africa. After three days in the hot climate there, she developed tiredness and fatigue with profuse vomiting. In hospital, routine bloods showed low sodium less than 100 mmol/l and raised potassium at 5.9 mmol/l. Other routine blood parameters were normal and random cortisol was low at 136 nmol/l.

Using intravenous normal saline, she was started on gradual sodium replacement aiming at 8 mmol/l correction per day with daily electrolyte monitoring. The sodium improved to 130 mmol/l over 5 days. Over the next week she developed dysarthria and ataxia with rigidity and tremor. MRI showed faint symmetrical change laterally in the basal ganglia that were not felt to be significant.

With a deteriorating condition and no clear diagnosis, she was flown back to the UK as an emergency for assessment at our tertiary neurological centre.

She had been diagnosed with coeliac disease at the age of 14 and maintained close dietary control. Two months prior to her holiday, she was found to have hypothyroidism and had been started on thyroxine 25 mcg once a day. There was no other past medical or surgical history.

On examination she was slim and under-nourished with a noticeable tan. She was afebrile and pulse and blood pressure were normal. Cardiovascular, respiratory and abdominal examinations were normal. Glasgow coma scale was 14/15. She was unable to speak, but was responding to questions with grunting and communicating with great difficulty using a finger-board.

The cranial nerves and fundus were normal. Tone was uniformly increased with cogwheel rigidity and tremor. Remaining neurological examination was normal.

The blood tests were normal on admission including the hepatic and renal functions. Sodium was 135 and potassium 3.9.

Thyroid functions were normal and thyroid peroxidase antibody was negative. The serum cortisol was low at 83 nmol/l and glucose was normal. A synacthen test was diagnostic of Addison's disease and her adrenal antibodies were positive. So, Addison's disease, exacerbated by dehydration and vomiting, had been the cause for her hyponatraemia.

Chest Xray, ECG and CT Scan of brain were normal.

At this stage, a metabolic insult to the brain due to osmotic shift during correction of hyponatraemia was thought of clinically and an MRI scan was requested.

**Figure 1:**
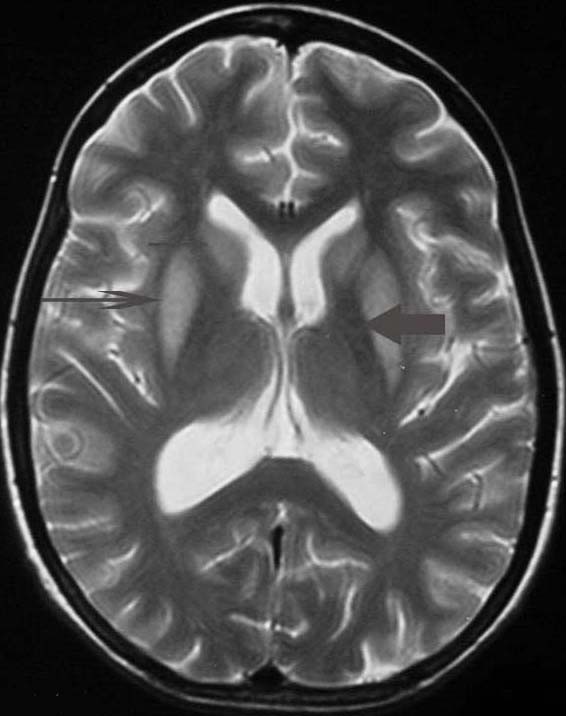
T2 weighted magnetic resonance scan image showing bilaterally symmetrical hyperintensities in Caudate nucleus (small, thin arrow), Putamen (long arrow), with sparing of Globus Pallidus (broad arrow), suggestive of Extrapontine myelinolysis.

MRI following transfer showed symmetrical hyperintensity on the T2-weighted images within the caudate and putamen (Fig 1) and in FLAIR T2 images (Fig 3). There were absolutely no changes within the pons in T2-weighted images (Fig 4). These changes did not show marked progression in comparison with the MR done soon after the metabolic insult. There were no changes within the globus pallidus, cerebellum or within cerebral white matter.

She was treated with hydrocortisone 200 mg tds intravenously and thyroxine 100 mcg daily. Fludrocortisone was subsequently added. Her neurological symptoms were controlled with Baclofen 20 mg bd and LevoDopa/carbidopa 100/25 mg tds. She improved markedly and levodopa therapy was no longer required after day 10. Baclofen was also withdrawn and she returned to work within 2 months.

**Figure 2:**
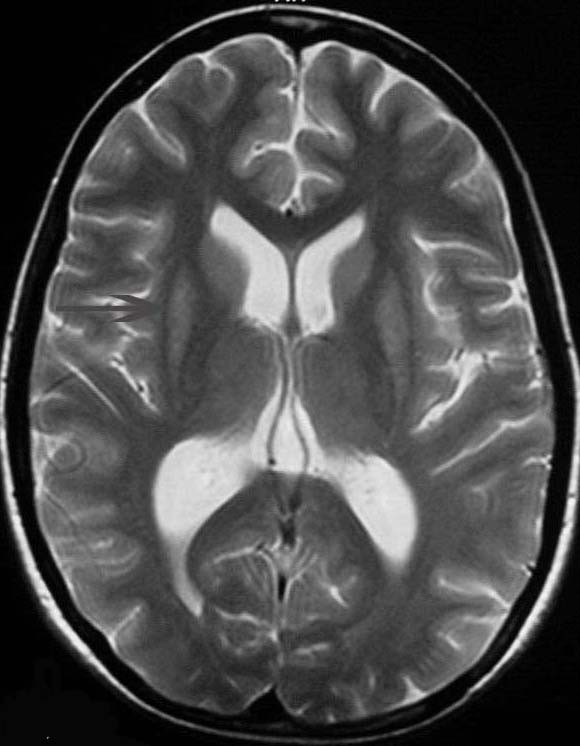
T2 weighted magnetic resonance scan image after two months showing marked improvement, mainly in putamen (long arrow).

**Figure 3:**
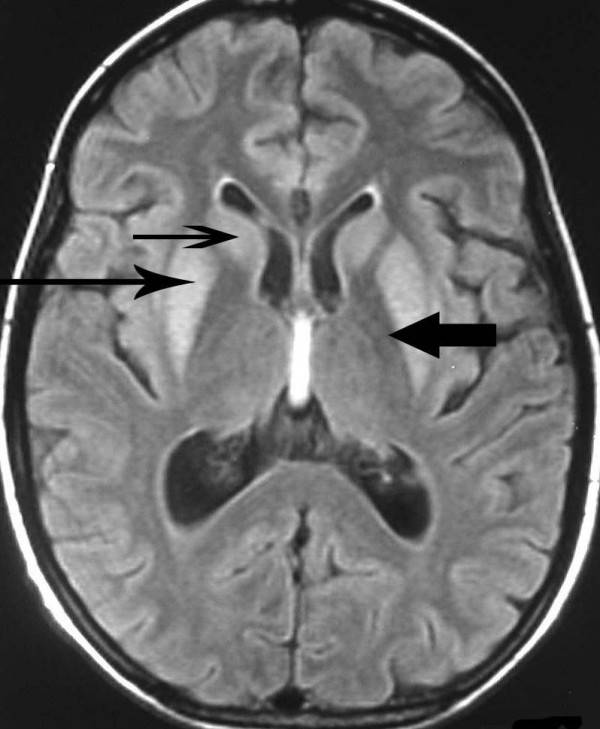
T2 weighted FLAIR images of magnetic resonance scan showing bilaterally symmetrical hyperintensities in Caudate nucleus (small, thin arrow), Putamen (long arrow), with sparing of Globus Pallidus (broad arrow), suggestive of Extrapontine myelinolysis.

**Figure 4:**
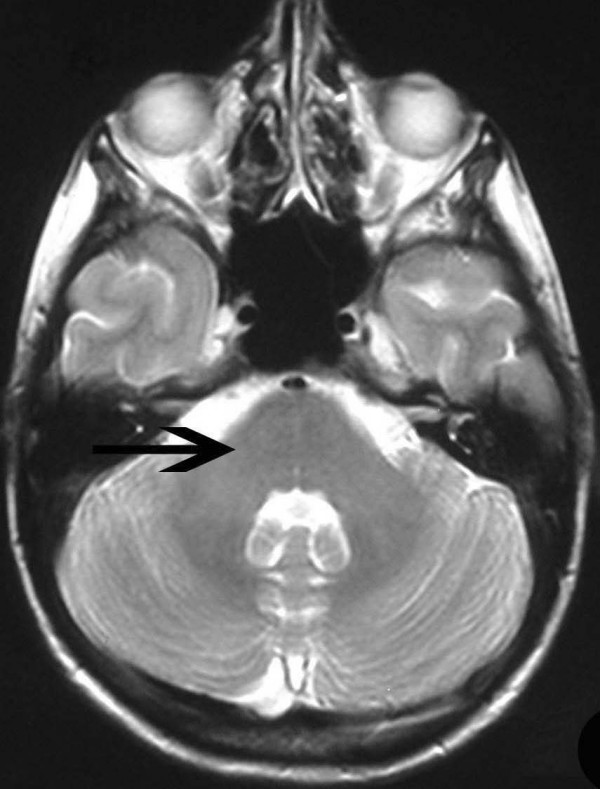
T2 weighted magnetic resonance scan image of pons showing absolutely no changes.

Follow-up MRI 2 months later showed significant resolution of the basal ganglia changes (Fig 2).

## Conclusion

The patient presented with features suggestive of parkinsonism, which had developed acutely following correction of severe hyponatraemia. The history and examination excluded the majority of conditions in Table 1.

Central pontine myelinolysis (CPM) may follow correction of hyponatraemia and generally presents with tetraparesis and brain stem dysfunction that may include pontine dysfunction, pseudobulbar palsy and a 'locked-in' syndrome. EPM has been described in 10% of patients with CPM [1], however extra-pyramidal symptoms are a rarity because these are masked by pyramidal tract and brain stem dysfunction.

In our patient, features of CPM were absent. There was no depression in conscious level, 'locked-in' syndrome, tetraplegia, pseudobulbar palsy, nystagmus or cranial nerve palsy. Positive findings included tremor, decreased speech and oro-bucco-lingual movements. This constellation of findings, which may be attributed to striatal dysfunction, has been described in isolated cases of EPM [2]. EPM may involve the basal ganglia, thalamus, cerebellum and subcortical white matter. Within the zone of demyelination, blood vessels, neurons and axis cylinders are largely spared and inflammation is absent [3].

In previous reports, the rapid correction of serum sodium to normal, or change in serum sodium greater than 25 mmol/l within 48 hours, particularly when associated with hypoxia, were linked to myelinolysis [4]. Recent reports suggests hyperosmolality and rapid osmotic shifts of fluid that cause hypernatremia [5], in addition to hypoglycemia, hypokalemia and azotemia, causes myelinolysis rather than absolute serum sodium level.[3] Pre-existing malnourishment, alcoholism, drug misuse, Addison's disease and immuno-suppression all increase the risk of myelin damage[2].

Symptoms and radiographic changes typically occur between 7 and 14 days after acute osmotic shift[3].

Osmotic myelinolysis typically cause trident-shaped signal change within the pons on T2-weighted images[8]. There is sparing of the cortico-spinal tracts and the tegmentum. This radiological appearance may persist despite clinical improvement[7]. Extrapontine changes have been described in the basal ganglia, white matter and cerebellum. Typically there is pallidal sparing[8]. In this case, the history of low sodum with subsequent correction, the clinical syndrome and the basal ganglia changes with sparing of the globus pallidus were strong pointers to the diagnosis of EPM, despite the absence of pontine changes. Bilateral signal change confined to the putamen, globus pallidus or thalamus may be seen in perinatal hypoxic-ischaemic injury and in Wilson's disease. There is predominant pallidal involvement in carbon monoxide poisoning and metabolic disorders such as Leigh disease. Patchy multifocal signal change may be seen in the basal ganglia in ADEM, encephalitis including Creutzfeldt-Jakob, lupus vasculitis or indeed any vascular disease involving perforators, deep venous occlusion (typically thalamic) and the mitochondrial cytopathies [6]. In recent writings on this subject, favourable outcome was observed for myelinolysis in patients with adrenal failure in the background of Polyglandular autoimmune syndrome and prolactinoma [9,10]. The destruction of the myelin alone without involvement of the axis cylinders or blood vessels because of rapid osmotic shift might explain our patients prompt clinical and radiological improvement.

## Competing interests

The author(s) declare that they have no competing interests.

## Authors' contributions

JS, AD and HAK reviewed the existing literature. JS drafted the manuscript which was edited by AD and HAK. A detailed Neuro-radiological discussion was added by AD. JS and HAK contributed to the thoughtful care of the patient presented, reviewed the manuscript and contributed to the writing. All authors read and approved the final manuscript.

## Pre-publication history

The pre-publication history for this paper can be accessed here:



## Figures and Tables

**Table 1:** Differential Diagnosis of Extrapyramidal syndrome.

**Hereditary disorders**
• Wilson's disease
• Huntington's disease
**Vascular parkinsonism**
• Infarcts
• Vasculitis
**Structural lesions**
• Gliomas
• Meningioma
**Hypoxic injury Infections**
• ADEM(acute demyelinating encephalomyelitis)
• Postencephalitic
• Creutzfeldt-jakob disease
**Metabolic**
• Central and Extrapontine myelinolysis
• Hypoparathyroidism
**Mitochondrial cytopathies Posttraumatic Drugs**
phenothiazines, butyrophenones, thioxanthenes Metoclopramide, methyldopa, reserpine, verapamil, flunarizine, amiodarone.
**Toxins**
carbon monoxide, cyanide, mptp, manganese.
